# Hospital-Onset Bloodstream Infections: Progressive Shift from Methicillin-Resistant *Staphylococcus aureus* to Methicillin-Susceptible *Staphylococcus aureus* over 11 Years

**DOI:** 10.3390/jcm15072645

**Published:** 2026-03-31

**Authors:** Silvia Boni, Marina Sartini, Filippo Del Puente, Giulia Adriano, Mauro Nelli, David Usiglio, Alessio Carbone, Gianluca Ottria, Marcello Feasi, Elisa Schinca, Nicoletta Bobbio, Emanuele Pontali, Maria Luisa Cristina, on behalf of the Galliera Infection Control Working Group (GICoWoG)

**Affiliations:** 1 Department of Infectious Diseases, Galliera Hospital, 16128 Genoa, Italy; silvia.boni@galliera.it (S.B.); filippo.del.puente@galliera.it (F.D.P.); marcello.feasi@galliera.it (M.F.); nicoletta.bobbio@galliera.it (N.B.); 2 Department of Health Sciences, University of Genoa, 16132 Genoa, Italy; sartini@unige.it (M.S.); alessio.carbone@galliera.it (A.C.); gianluca.ottria@unige.it (G.O.); elisa.schinca@unige.it (E.S.); cristinaml@unige.it (M.L.C.); 3 Operating Unit Hospital Hygiene, Galliera Hospital, 16128 Genoa, Italy; 4 Hospital Infection Control Office, Galliera Hospital, 16128 Genoa, Italy; giulia.adriano@galliera.it; 5 Health Directorate Galliera Hospital, 16128 Genoa, Italy; mauro.nelli@galliera.it; 6 Department of Laboratory and Microbiological Analysis, Galliera Hospital, 18128 Genoa, Italy; david.usiglio@galliera.it; 7 Department of Medicine, Division of Medical Oncology, Galliera Hospital, 16128 Genoa, Italy

**Keywords:** *Staphylococcus aureus*, epidemiology, methicillin resistance, hospital-onset bacteraemia, infection prevention

## Abstract

**Background**: *Staphylococcus aureus* bloodstream infections cause substantial hospital mortality (20–50%). Surveillance programs primarily focus on methicillin-resistant *S. aureus* (MRSA) as a quality indicator, while comprehensive data comparing MRSA and methicillin-susceptible *S. aureus* (MSSA) hospital-onset bacteremia remain limited outside the UK, the USA, and Canada. This study aims to evaluate incidence trends of all hospital-onset *S. aureus* bloodstream infections (HO-SABSIs, defined as positive blood cultures ≥ 48 h post-admission) from 2013 onwards, comparing MRSA and MSSA incidence patterns and their associated mortality rates in an Italian tertiary-care hospital through surveillance data. **Methods**: Retrospective cohort study of all HO-SABSIs from January 2013 to December 2023 at a 458-bed Italian tertiary hospital. Primary outcome was incidence density (cases per 1000 patient-days) stratified by methicillin resistance. Standardized Infection Ratios (SIRs) were calculated using 2013–2015 and 2013–2019 as baseline periods. Temporal trends were analyzed using negative binomial regression. **Results**: Among 414 HO-SABSI cases, 183 (44.2%) were MRSA and 231 (55.8%) MSSA. MRSA incidence decreased significantly (−6.5% annually; IRR 0.935, 95%CI 0.883–0.991; *p* = 0.023) from 0.198 to 0.111 per 1000 patient-days. MSSA increased non-significantly (+4.9% annually; IRR 1.049, 95%CI 0.999–1.103; *p* = 0.056) from 0.103 to 0.239, surpassing MRSA after 2016 and peaking during COVID-19 (2021: SIR 2.41, 95%CI 1.33–4.37). MRSA cases had higher mortality than MSSA (40.6% vs. 27.5%; *p* = 0.005). **Conclusions**: MSSA has replaced MRSA as the predominant hospital-onset *S. aureus* bacteremia pathogen since 2016, with sustained elevation during and after the COVID-19 pandemic. Current MRSA-focused surveillance misses the majority of preventable staphylococcal bacteremias. Comprehensive hospital-onset *S. aureus* surveillance encompassing both phenotypes is essential for accurate epidemiological assessment and infection prevention.

## 1. Introduction

*Staphylococcus aureus* (SA) is a Gram-positive bacterium posing a substantial global health burden. It is the leading bacterial cause of death worldwide, with an estimated 300,000 annual fatalities and a case fatality rate ranging from 15% to 30% [1,2,3]. SA bloodstream infections (SABSIs) represent a major clinical manifestation, ranking as the second most common bloodstream infection and a significant source of morbidity and mortality, with reported fatality rates of 20% to 50% [4]. SABSIs are consistently reported among the leading causes of hospital-acquired bacteremias [5,6].

Antimicrobial resistance complicates this challenging condition, representing a global public health crisis. Methicillin-resistant *Staphylococcus aureus* (MRSA) is a critical pathogen within this context, identified as the second most common cause of antibiotic-resistant infection in Europe and responsible for over 50% of multidrug-resistant infections among US hospitalized patients in 2017 [7]. Consequently, MRSA surveillance programs have been widely implemented. The European Multinational Program coordinated by the European Centre for Disease Prevention and Control (ECDC) relies on national systems [8]. England’s National Health Service (NHS) pioneered mandatory MRSA bacteremia surveillance in 2001, expanding to include methicillin-susceptible SA (MSSA) bacteremia in 2011 [9]. Similar integrated surveillance programs for both MRSA and MSSA, focusing on incidence, risk factors, and prevention strategies, have since been adopted in the USA and Canada [7,10]. In Italy, a national system for healthcare-associated infection (HAI) surveillance, established in 2019 through the Ministry of Health’s National Centre for Disease Prevention and Control (CCM), includes specific objectives for robust MRSA infection surveillance, particularly bloodstream infections, to support the National Action Plan on Antimicrobial Resistance (PNCAR) [11]. Despite these efforts, comprehensive surveillance data evaluating trends and comparing the incidence of both MRSA and MSSA bacteremias remain limited outside reports from the UK, Canada, and the USA [7,9,10]. This gap exists partly because hospital-onset (HO) MRSA bacteremia is often prioritized as a surrogate for invasive SA disease risk and a quality indicator. For instance, in the USA, HO-MRSA bacteremia is included in the Centers for Medicare & Medicaid Services’ Hospital-Acquired Condition Reduction Program via the National Healthcare Safety Network (NHSN), while MSSA bacteremia is not. The NHSN also provides a Standardized Infection Ratio (SIR) specifically for MRSA, adjusting for community-onset prevalence and hospital factors [12].

The most recent and robust incidence data for MRSA and MSSA bacteremias in Italy comes from an analysis of EARS-Net data published in Euro Surveillance, which covers the period 2005–2018. In Italy, the percentage of MRSA among SABSIs decreased from 30.2% in 2005 to 16.3% in 2018, while the total number of SABSIs increased by 57%. Specifically, MSSA BSIs increased by 84%, while those due to MRSA decreased by 31% over the same period. These data reflect a national and European trend, with an increase in the healthcare burden due to MSSA despite the declining proportion of MRSA [9]. Italian regional studies confirm the decreasing prevalence of MRSA in BSIs in recent years, with recent MRSA percentages ranging between 16% and 33% of SABSIs, depending on the hospital and geographic context [13,14]. However, the prevalence of MSSA is increasing, and now represents the majority of SABSIs.

To address the abovementioned surveillance gaps, HO bacteremia (HOB), defined by positive blood cultures obtained >48 h after admission, has emerged as a potential useful surveillance approach. Unlike traditional indicators focused on specific pathogens (e.g., MRSA) or infection sources (e.g., Central Line Associated Bloodstream Infection—CLABSI), HOB captures all hospital-acquired bloodstream infections through automated microbiological surveillance, potentially providing a more comprehensive picture of preventable infections including both MRSA and MSSA bacteremias [15,16]. Several reports suggest advantages of HOB surveillance over traditional methods like CLABSI tracking, noting that while CLABSI rates have declined over time, overall HOB remains a major cause of morbidity and mortality [5,6,17,18]. HOB, encompassing both central and peripheral lines sources, may offer a more sensitive indicator of preventable bloodstream infections [5,6,18].

Given the persistent burden of SABSIs, the limitations in current surveillance focusing primarily on MRSA, this observational study aims to evaluate incidence trends of HO-SABSIs from 2013 onwards, comparing MRSA and MSSA incidence patterns and their associated crude mortality rates in an Italian tertiary-care hospital through comprehensive surveillance data.

## 2. Materials and Methods

### 2.1. Study Design, Setting, and Data Collection

We conducted a retrospective cohort study of all HO-SABSIs occurring between 1 January 2013 and 31 December 2023, at a nationally renowned tertiary-care hospital in northern Italy. The 458-bed facility operates across three buildings with primarily 3–4 bedrooms, with over 15,000 inpatient admissions and more than 8600 outpatient/day-surgery procedures annually. The hospital is organized by treatment intensity levels.

HO-SABSI cases were identified using Centers for Disease Control and Prevention (CDC) criteria, defined as ≥1 positive blood culture for *S. aureus* obtained ≥48 h after hospital admission [19]. All positive blood cultures obtained during the study period were considered. The identification of *S. aureus* occurred by means of the BD Phoenix™ system (Becton, Dickinson and Company, Franklin Lakes, NJ, USA). Methicillin resistance was determined by standard microbiological susceptibility testing performed by the hospital laboratory using the BD Phoenix™ system. *S. aureus* represent 9–13% of all pathogens isolated from blood cultures at our hospital. Each patient was counted once even if multiple sets of blood cultures were positive within the same hospitalization episode.

For each confirmed case, we collected: patient sex and age; admission and discharge dates; patient outcome (survival or death); ward location at bacteremia onset; methicillin resistance status (categorized as MRSA or MSSA); presence of intravascular access devices (central venous catheter [CVC], peripherally inserted central catheter [PICC], or midline catheter); and date of positive blood culture.

### 2.2. Outcomes and Infection Metrics

The primary outcome was incidence density of HO-SABSIs, calculated as cases per 1000 patient-days and stratified quarterly and annually by MRSA/MSSA status. We additionally calculated the Standardized Infection Ratio (SIR) [20] for MRSA and MSSA bacteremias using two distinct baseline periods: for long-term assessment, 2013–2015 served as the initial benchmark to compute expected events for 2016–2023, with expected events calculated as the mean annual incidence rate from the baseline period multiplied by patient-days in each subsequent year; to evaluate pandemic impact specifically, 2013–2019 provided a pre-pandemic benchmark for assessing COVID-19 impact during 2020–2023, using the same calculation method. SIR values > 1.0 indicated observed events exceeding predictions, while values < 1.0 indicated fewer events than predicted. Crude mortality SIRs were similarly calculated for MRSA- and MSSA-associated deaths.

### 2.3. Statistical Analysis

Continuous variables are presented as mean ± standard deviation and median (range), while categorical variables are expressed as counts and percentages. Group comparisons employed Chi-square tests for categorical variables and Wilcoxon rank-sum tests for continuous variables with non-normal distributions. Temporal trends in infection incidence were analyzed using negative binomial regression after confirming overdispersion (α) and superior model fit versus Poisson regression through lower Akaike Information Criterion (AIC) and Bayesian Information Criterion (BIC) values. All SIRs are reported with 95% confidence intervals. Statistical significance was defined as *p* < 0.05 (two-tailed), with analyses performed in Stata/SE 19.0 (StataCorp LP, College Station, TX, USA).

## 3. Results

During the 11-year study period (2013–2023), 414 HO-SABSIs were identified, including 183 MRSA (44.2%) and 231 MSSA (55.8%) cases. Patient demographics revealed a male predominance without significant sex-based differences between MRSA and MSSA groups. MRSA patients were significantly older than MSSA patients (median age 80 vs. 75 years, *p* < 0.001). Intravascular devices were associated with 16.7% of infections, with no significant difference between MRSA and MSSA cases (Table 1).

**Figure 1:**
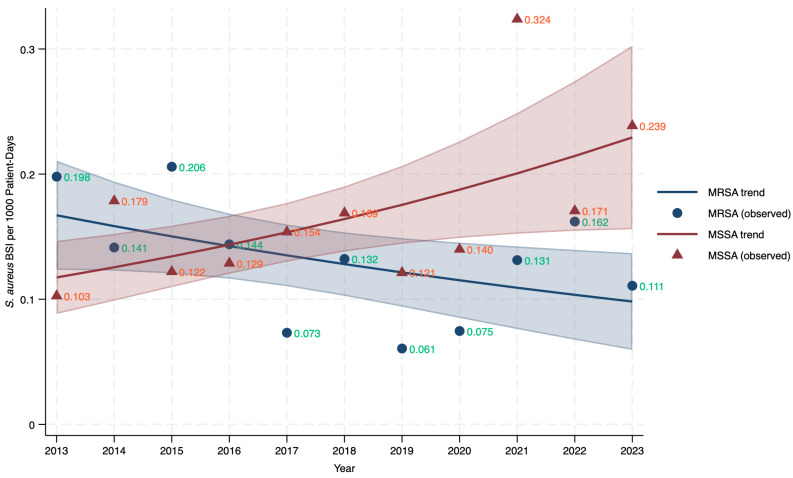
Annual incidence of hospital-onset methicillin-susceptible *S. aureus* (MSSA) and methicillin-resistant *S. aureus* (MRSA) bloodstream infections.

Annual incidence trends demonstrated divergent trajectories (Figure 1). MSSA incidence increased from 0.103 to 0.239 per 1000 patient-days (+132%), peaking at 0.324 during the COVID-19 pandemic (2021). Conversely, MRSA incidence declined from 0.198 to 0.111 per 1000 patient-days (−44%), with MSSA surpassing MRSA after 2016 (*p* < 0.05 from 2021 onward). Negative binomial regression confirmed a significant 6.5% annual reduction in MRSA incidence (IRR 0.935, 95% CI 0.883–0.991; *p* = 0.023) versus a non-significant 4.9% annual increase in MSSA (IRR 1.049, 95% CI 0.999–1.103; *p* = 0.056).

**Figure 2:**
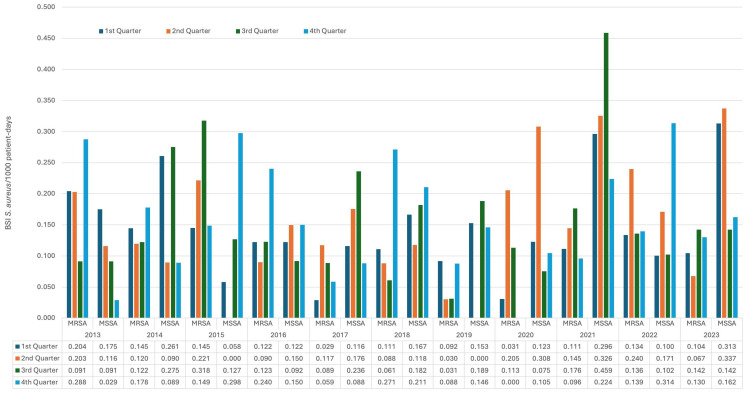
Incidence of SABSI per 1000 patient-days by quarter and year (methicillin-susceptible *S. aureus* (MSSA) vs. methicillin-resistant *S. aureus* (MRSA)).

Quarterly analysis revealed distinct temporal patterns (Figure 2): MSSA peaked in Q3-2021 (0.459/1000 patient-days), with secondary surges during the COVID-19 pandemic periods. MRSA demonstrated earlier peaks (Q3-2015: 0.318/1000 patient-days), with post-2019 values consistently below historical levels despite transient increases during COVID-19 pandemic quarters.

**Figure 3:**
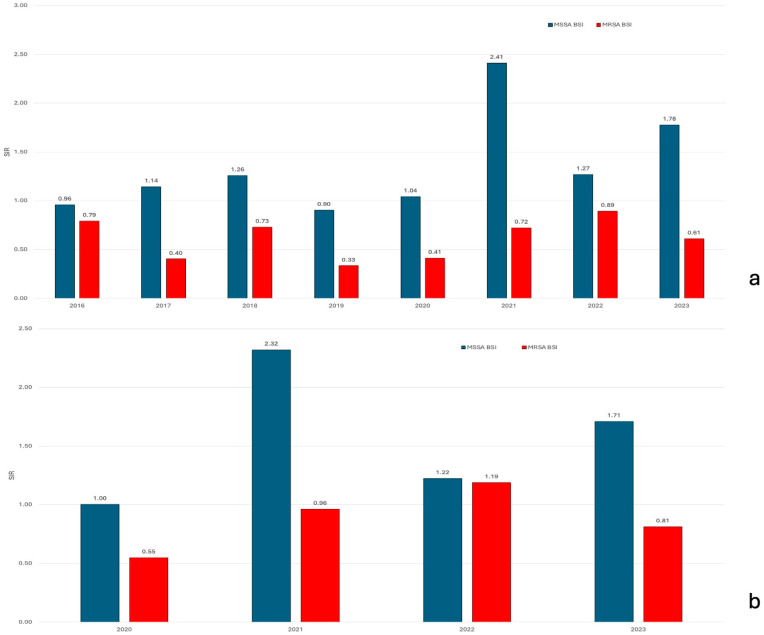
Standardized Infection Ratios (SIRs) for hospital-onset methicillin-susceptible *S. aureus* (MSSA) and methicillin-resistant *S. aureus* (MRSA) bacteremias. (**a**) Baseline period 2013–2015, showing 2016–2023. (**b**) Baseline period 2013–2019, showing pandemic years 2020–2023. SIR = 1.0 indicates observed incidence equal to baseline expectation.

Standardized Infection Ratios (SIRs) using 2013–2015 as baseline confirmed these trends (Figure 3a). The MRSA SIR remained below 1.0 throughout (range: 0.33–0.89), indicating sustained reduction. The MSSA SIR exceeded 1.0 from 2017 onward, peaking at 2.41 in 2021 and remaining numerically elevated through 2023.

When standardized to the pre-pandemic period (2013–2019), the MSSA SIR remained statistically significantly elevated during the COVID-19 pandemic years (2021: 2.32; 2023: 1.71), while the MRSA SIR fluctuated near baseline (Figure 3b).

**Figure 4:**
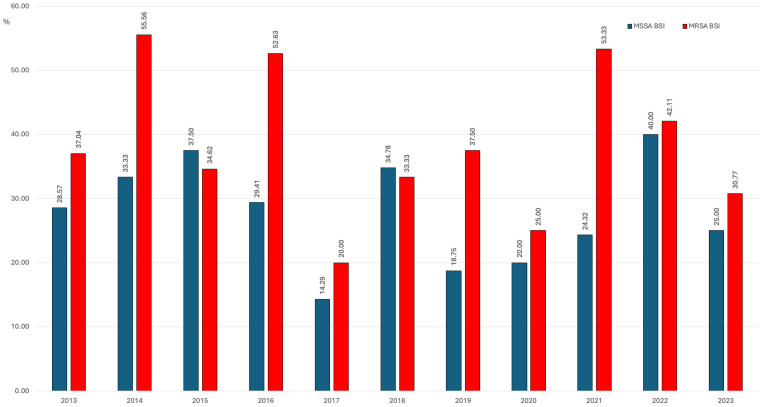
Percentage trend of mortality for BSI due to methicillin-susceptible *S. aureus* (MSSA) and methicillin-resistant *S. aureus* (MRSA).

Outcome data were available for 409 patients (98.8%; 5 patients had missing outcome data). MRSA cases had significantly higher mortality than MSSA cases (40.6% vs. 27.5%; *p* = 0.005). Mortality peaked for MRSA in both 2014 (56.5%) and 2021 (53.3%), while MSSA mortality was highest in 2022, at 40.0% (Figure 4).

**Figure 5:**
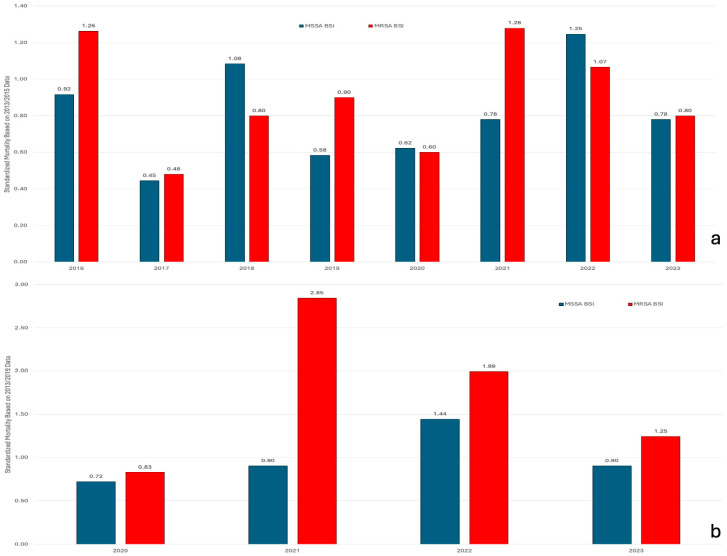
Standardized mortality for hospital-onset methicillin-susceptible *S. aureus* (MSSA) and methicillin-resistant *S. aureus* (MRSA) bacteremias. Panel (**a**): standardized to 2013–2015 baseline. Panel (**b**): standardized to pre-pandemic period (2013–2019). SIR = 1.0 indicates observed incidence equal to baseline expectation.

Mortality SIR analysis revealed excess MRSA deaths in 2016 and 2021 when standardized to 2013–2015 (Figure 5a), with a pronounced 2021 mortality surge for MRSA when standardized to 2013–2019 (SIR 2.85). MSSA mortality exceeded expectations in 2018 and 2022 under both standardization methods (Figure 5b).

## 4. Discussion

This 11-year analysis reveals a significant epidemiological transition in HO-SABSIs within an Italian tertiary-care hospital setting. Our data demonstrate a clear shift in pathogen dominance, with MSSA surpassing MRSA in incidence since 2016—a trend that intensified during the COVID-19 pandemic [21].

Statistical modeling confirmed a significant annual decrease in MRSA incidence (−6.5%; *p* = 0.023) while contrasting with a non-significant upward trajectory for MSSA (+4.9% annually; *p* = 0.056). Standardized Infection Ratio (SIR) analysis robustly validated these findings, showing MRSA consistently below expected levels while MSSA exceeded predictions from 2017 onward, peaking at 2.41 times baseline during the COVID-19 pandemic [22].

Despite declining incidence, MRSA infections carried substantially higher mortality (40.6% vs. 27.5% for MSSA; *p* = 0.005), underscoring MRSA’s persistent clinical threat. The observed epidemiological shift, with MSSA surpassing MRSA incidence since 2016 and peaking during the COVID-19 pandemic, represents a significant finding that challenges the current surveillance paradigm focused predominantly on MRSA. The divergent temporal trajectories—with MRSA showing a sustained decline (IRR 0.935; *p* = 0.023) while MSSA demonstrated a non-significant upward trend (IRR 1.049; *p* = 0.056)—suggest that these two phenotypes may have distinct epidemiological drivers that warrant separate surveillance and investigation [7,23].

While the drivers of this epidemiological shift were not formally investigated and fall outside the scope of this observational study, several factors may have contributed to the divergent trends observed. Similar trends have been reported by a European report [9] collecting data from 573,951 SABSI cases (96,918 MRSA and 477,033 MSSA bacteremias) from 25 countries [9]. Some of the potentially impacting factors could include variations in antibiotic prescribing practices, the evolution of device management bundles, the increase in the use of blood culture sets as a result of diagnostic stewardship efforts during the study periods, the evolution of infection control strategies primarily targeting MRSA, and the studied populations’ differences in clinician approaches to managing *S. aureus* infections, as evidenced by substantial practice variation in international surveys [9,22]. Actually, such activities were sometimes carried out at our hospital, specifically, (1) an intensified training and informative campaign on hand hygiene in all departments in 2016; (2) training sessions for all nurses for CVC/PICC and peripheral lines management in 2016; (3) expansion of MRSA nasal carriage screening and decolonization to all candidates to instrumented surgery in 2023. Unfortunately, it is not possible to clearly and definitely correlate such activities with the observed trends of MRSA/MSSA.

Methodologically, this study demonstrates that comprehensive surveillance of all HO-SABSIs, rather than selective MRSA monitoring, is essential to capture the full burden of hospital staphylococcal disease. Our HOB-based surveillance approach proved more sensitive than traditional methods, identifying infections that would not have been captured through CLABSI monitoring alone, given that over 80% of HO-SABSIs occurred without central lines.

The observed epidemiological patterns—particularly the MSSA increase during and after the COVID-19 pandemic—highlight critical gaps in our understanding of MSSA transmission dynamics in healthcare settings. The persistence of elevated MSSA rates in the post-pandemic period (SIR 1.71 in 2023 when standardized to 2013–2019) suggests that factors beyond temporary pandemic-related disruptions may be contributing to this trend, as is also observed at the European level [9].

Limited or no information is available on specific MSSA preventive measures. It can be supposed that the greater focus on MRSA surveillance and prevention has not been properly balanced by research in specific MSSA prevention measures. MSSA bacteremias clearly have a distinct epidemiological pattern, which is not yet precisely defined, and one in which the management of peripheral lines rather than central ones may play a role.

Study limitations include the single-center retrospective design and the absence of molecular typing data that could have provided insights into clonal dynamics. Nevertheless, the 11-year observation period and comprehensive case capture through automated surveillance provide robust epidemiological data.

These findings underscore a diffuse and urgent need for surveillance systems that encompass both MRSA and MSSA to accurately characterize the evolving epidemiology of HO-SABSIs [24,25]. The observed MRSA-to-MSSA shift has important implications for infection prevention priorities and resource allocation. Future research should focus on understanding the mechanisms driving MSSA emergence in healthcare settings and investigating whether current prevention strategies, largely designed around MRSA, require adaptation to effectively address MSSA. The implementation of comprehensive HOB surveillance, as demonstrated in this study, represents a practical approach for monitoring these trends and should be considered for broader adoption in national surveillance programs.

## Figures and Tables

**Table 1:** Distribution of bacteremias by year and patient characteristics.

	All Patients		MRSA		MSSA		*p*-Value
	(*N* = 414)		(*N* = 183)		(*N* = 231)		
	*N*	%	*N*	%	*N*	%	
Sex: Males	237	42.75	113	61.75	124	53.68	n.s.
Intravascular Device							n.s.
CVC/PICC	55	13.29	28	15.3	27	11.69	
Midline	14	3.38	8	4.37	6	2.6	
No device	345	83.33	147	80.33	198	85.71	
Year of isolation							<0.001
2013	41	9.9	27	14.75	14	6.06	
2014	43	10.39	19	10.38	24	10.39	
2015	43	10.39	27	14.75	16	6.93	
2016	36	8.70	19	10.38	17	7.36	
2017	31	7.49	10	5.46	21	9.09	
2018	41	9.90	18	9.84	23	9.96	
2019	24	5.80	8	4.37	16	6.93	
2020	23	5.56	8	4.37	15	6.49	
2021	52	12.56	15	8.20	37	16.02	
2022	39	9.42	19	10.38	20	8.665	
2023	41	9.90	13	7.10	28	12.12	
Outcome *							0.018
Deceased	136	33.25	73	40.55	63	27.51	
Discharged	273	66.75	107	59.45	166	72.49	
Age							
Mean (range)	75	(17–99)	78	(23–99)	73	(17–99)	
Median (IQR)	78	(68–86)	80	(73–87)	75	(64–85)	<0.001

n.s.: not significant; * Three patients in the MRSA group and two patients in the MSSA group experienced two different BSIs during the same hospitalization, so the outcome was only counted once. Legend: CVC: central venous catheter; MRSA: methicillin-resistant *S. aureus*; MSSA: methicillin-susceptible *S. aureus*; PICC: peripherally inserted central catheter.

## Data Availability

The raw data supporting the conclusions of this article will be made available by the authors on request.

## Abbreviations

The following abbreviations are used in this manuscript:

MRSA	methicillin-resistant *S. aureus*
MSSA	methicillin-susceptible *S. aureus*
CLABSI	central line-associated bloodstream infection
SABSI	*S. aureus* bloodstream infection
HO	hospital onset
SIR	standardized infection ratio
